# Milk

**Published:** 1859-04

**Authors:** 


### MILK,

Fresh, pure, luscious, cream and all, from farm-fed cows, is served on our table every day, within a dozen hours after it is drawn from its natural fountain. What a countless multitude of Gothamites envy our good fortune, and how the question leaps from the lips "How do you get it?" Don't be in a hurry. We will tell you by degrees, not only how we get it, but how you may get it, and at common milk cart prices for the adulterated article; swill one half, water one quarter, milk "a trace," as a chemist would say.

A New Yorker does not know what pure milk is, theoretically or gustatorially. In the first place, nobody would drink pure milk willingly, except babies, pigs, and such like. Within our remembrance, we never drank but one fill of it, and that was over twenty years ago. We have vivid recollections of its lusciousness even now, by virtue of the peculiar circumstances and surroundings of the case. We were beyond the boundaries of civilzation. We were literally among the savages. We had been travelling across the wilderness for a long, long time, day in and day out, our provisions were exhausted, no game to be seen in those wild, wild regions, and weary and faint, at close of day, we suddenly confronted an Indian encampment, and making our way to the wigwam of the chief, who was at once recognized by his dress and presence, sitting at the door of his tent, we signed that we were hungry. Instantly his maiden daughter, as straight as an arrow, and with the graceful spring of youth, ran to the herd, and in a few moments presented a vessel foaming and brimming full to our thirsty lips, and such a quaff of nectar we never had before or since, despite of its natural warmth, and the multitude of little scales and other things, from the cows teats and elsewhere, which were swimming in discouraging abundance on the surface. We soon found it was no use in blowing them aside, and we comforted ourself in the reflection, that although these particles were not exactly milk itself, they were pretty *near* milk, at all events! and down they went; the diversion from this very slight circumstance being, the contemplation of the beautiful young creature before us—for we were then young.

In ordinary weather, milk remains fresh and pure for about three hours after the milking, when it begins to decompose, that is, begins to putrify, and hence is no longer "pure" milk, no more than meat is fresh or pure after it has begun to decay. These being the facts of the case, there is sound philosophy in recommending milk "warm from the cow." Hence we have seen in St. James Park, London, within a few yards of the palace of the Queen, a fine healthful looking cow tied to a tree, and nurses coming up in their turn, with little cups to be filled from the teat, to be drank by the children and infants under their charge.

Science, and art, and invention, have led to several plans of securing milk in its purity to that large class of persons who live in towns and cities and on the sea, to whom a cow is an impossibility. SOLIDIFIED MILK is one result, and is prepared as follows: premising that of every thousand pints of milk, as it comes from the cow, eight hundred and sixty-one pints are simple water, this water is evaporated away in the manner of making sugar out of cane juice, or "sugar water," when a thick doughy substance is left, which on being dried, chrystalizes, and becomes a granulated, yellowish, creamy tinted, dry powder, it is then put in tin cans, covered tight, and remains fit for use at once, or for months after, having however finely pulverized loaf sugar mixed with it, to preserve it longer. Hence solidified milk is not pure milk, it is sugar and milk. It is prepared for use by re-adding the amount of water which was at first taken from it, or it is stirred in tea and coffee, until whitened to the taste of each person. This milk is prepared by a New York Company, in the midst of an agricultural district, out of convenient reach of conveyance to the city, where they are glad to sell it for two or three cents a quart, as it comes from the cow.

CONDENSED MILK is another form of more recent introduction, the discovery of Gail Borden, a man of great reflective powers, and of indomitable energy. He has spent long years of time and anxious thought, with many thousands of dollars, before he succeeded in bringing it to its present perfection of process, *and* in persuading the people that it was a reality and not an imposition. Mr. Borden's establishment is also in an agricultural region, where the milk would otherwise find no market. The farmers bring it while yet warm, it is then deprived of a large part of its water, which leaves it of the thickness of syrup or molasses, it is then placed in a cool vessel surrounded with ice, sent to New York, and delivered from these vessels at the doors of those who use it. Some of the ocean steamers take enough for a voyage out and back, keeping it in an ice chest, and it is apparently as good on the day of return, as on the day of departure. Nothing is mixed with it, not even sugar. It is essentially a pure milk. All the preparation needed for using it, is simply to stir it in the tea and coffee. If it is to be drunk or used for cooking purposes, common cold water is to be stirred in, until it is reduced to the proper thinness. One pint of condensed milk with three pints of pure water, will make a thicker, richer milk than is to be had of any city milk dealer, and at a rate about the same that is paid for ordinary cart milk.

As long as one pint of condensed milk weighs twenty ounces, we are getting what we pay for, a less weight for a pint, is that much of a fraud.

There are two things which will oppose the introduction of condensed milk—1st. The servants will see at once its richness, and will use the concentrated condensed article, with the same measure and freeness as they use the common kind. 2nd. It requires more care in handling, than the generality of city mistresses will give to it, and being left to servants will be badly and wastefully used. The only care required is that the vessel containing the milk, shall remain in the refrigerator or other cool place, all the time, taking only enough from it, as will answer the occasion, for if it is taken to the kitchen or placed on table during the meals, the whole of it gets warm and sours, but even then, for all the purposes of sour milk, it is most admirable.

As pure milk is associated with fresh eggs, it may be well to state that while milk remains pure only for about three hours after it is taken from the cow, an egg is not fit to eat, is not " set" until about twelve hours after it has been laid.

We may as well complete this essay on milk, by stating that it is the only article of food yet known to chemists, which has in it all the elements of nutrition. Hence it is sufficient to sustain the child until it is weaned. No grown person could live and work, and thrive so long, on any one article of food. So wise and kind is that Providence which opens in the mother's bosom a fountain which contains all that the child requires for its fullest wants.

In every thousand pounds of milk there are of—

| | | |
|---|---|---|
| Water, | 861 | pounds, |
| Caseine or Cheese | 66 | " |
| Butter, | 38 | " |
| Sugar, | 29 | " |
| Salts, | 6 | " |

That is, the water quenches thirst, the cheese makes the flesh, the butter oils the skin and keeps it soft, lubricates the joints, and like the sugar, which makes the milk palateable, it affords material for warmth, by means of the respiration, while the fixed salts go to make up the bones as a frame work for the body. But when the child gets old enough to run about, it must have food which contains more of these phosphatic salts; if it does not, it becomes rickety, being proof conclusive, that milk was designed as the main food, only for the very young.

But to make this more directly practical to city readers, who are beginning in this April spring time, to yearn for the country, its milk, and butter and eggs, for themselves and their children, the first enquiry to be made when a "farm house" is found, should be, not whether they keep cows and hens, for they will point you at once to the finest, largest, sleekest animals you ever laid eyes on; and as for hens, the whole yard cackles and the little chickens come about your feet, so that it is difficult not to tread on them, but ask this pregnant question, —Do you get a good price for your milk, eggs and butter? or the more effectually to guard yourself from imposition, visit said farm houses *incog*, as a "middle man" or huckster, and price these valuable articles, the grass butter, the snowy eggs, the luscious milk, and act accordingly.

But while the "solidified" and "condensed" milk are admirable for their respective purposes, the Rockland County Association have gone a step farther, and propose to deliver milk twice a day during the summer, in a state as natural and pure as at the moment of the milking, except that it shall be of a refreshing coolness, and all this, at the common price of seven cents a quart. It is interesting to know how this thing is done.

It is delivered as is the condensed milk, while yet warm from the cow into the cans of the agent, who has these cans placed in cold water, while the milk in them is kept stirred by machinery until it is cold, he then locks each can, and places it in a box, surrounded with ice, but so as not to make it ice cold, for that also would decompose the milk, he then accompanies these cans to the city on the rail road, and on reaching their depot in Tenth street, the cans are unlocked, and multitudes of smaller ones holding two quarts each, are filled and locked, the names of the persons to whom these cans are to be delivered is painted on them. Each can lock has a duplicate key, the agent keeps one, the customer the other. The milking of last night is delivered for breakfast, that of the morning is delivered at sun down.

We may safely say, that until this company gets into full operation, and a large custom, we will very certainly have pure milk in its natural state, and fresh, placed on our tables, but with thousand of customers, the temptation to adulterate will most likely become too strong to resist. Meanwhile, those who patronize the association for the first year or two, may, for that time at least, with their children, luxuriate in pure, fresh milk, from farm house cows.

